# Outcomes of a Remote Cardiac Rehabilitation Program for Patients Undergoing Atrial Fibrillation Ablation: Pilot Study

**DOI:** 10.2196/49345

**Published:** 2023-12-14

**Authors:** Satish Misra, Karen Niazi, Kamala Swayampakala, Amanda Blackmon, Melissa Lang, Elizabeth Davenport, Sherry Saxonhouse, John Fedor, Brian Powell, Joseph Thompson, John Holshouser, Rohit Mehta

**Affiliations:** 1 Sanger Heart and Vascular Institute Charlotte, NC United States

**Keywords:** atrial fibrillation, behavior modification, cardiac rehabilitation, catheter ablation, exercise, remote exercise supervision, weight loss

## Abstract

**Background:**

Risk factor modification, in particular exercise and weight loss, has been shown to improve outcomes for patients with atrial fibrillation (AF). However, access to structured supporting programs is limited. Barriers include the distance from appropriate facilities, insurance coverage, work or home responsibilities, and transportation. Digital health technology offers an opportunity to address this gap and offer scalable interventions for risk factor modification.

**Objective:**

This study aims to assess the feasibility and effectiveness of a 12-week asynchronous remotely supervised exercise and patient education program, modeled on cardiac rehabilitation programs, in patients with AF.

**Methods:**

A total of 12 patients undergoing catheter ablation of AF were enrolled in this pilot study. Participants met with an exercise physiologist for a supervised exercise session to generate a personalized exercise plan to be implemented over the subsequent 12-week program. Disease-specific education was also provided as well as instruction in areas such as blood pressure and weight measurement. A digital health toolkit for self-tracking was provided to facilitate monitoring of exercise time, blood pressure, weight, and cardiac rhythm. The exercise physiologist remotely monitored participants and completed weekly check-ins to titrate exercise targets and provide further education. The primary end point was program completion. Secondary end points included change in self-tracking adherence, weight, 6-minute walk test (6MWT), waist circumference, AF symptom score, and program satisfaction.

**Results:**

The median participant age was 67.5 years, with a mean BMI of 33.8 kg/m^2^ and CHADs2VASC (Congestive Heart Failure, Hypertension, Age [≥75 years], Diabetes, Stroke/Transient Ischemic Attack, Vascular Disease, Age [65-74 years], Sex [Female]) of 1.5. A total of 11/12 (92%) participants completed the program, with 94% of expected check-ins completed and 2.9 exercise sessions per week. Adherence to electrocardiogram and blood pressure tracking was fair at 81% and 47%, respectively. Significant reductions in weight, waist circumference, and BMI were observed with improvements in 6MWT and AF symptom scores (*P*<.05) at the completion of the program. For program management, a mean of 2 hours per week or 0.5 hours per patient per week was required, inclusive of time for follow-up and intake visits. Participants rated the program highly (>8 on a 10-point Likert scale) in terms of the impact on health and wellness, educational value, and sustainability of the personal exercise program.

**Conclusions:**

An asynchronous remotely supervised exercise program augmented with AF-specific educational components for patients with AF was feasible and well received in this pilot study. While improvements in patient metrics like BMI and 6MWT are encouraging, they should be viewed as hypothesis generating. Based on insights gained, future program iterations will include particular attention to improved technology for data aggregation, adjustment of self-monitoring targets based on observed adherence, and protocol-driven exercise titration. The study design will need to incorporate strategies to facilitate the recruitment of a diverse and representative participant cohort.

## Introduction

Atrial fibrillation (AF) is one of the most common arrhythmias affecting adults, with a projected prevalence in the United States of 12.1 million by 2030. With nearly 5 million office visits and 700,000 emergency department visits annually, AF is associated with a significant burden on the health care system that is expected to grow in the coming years [1-3]. In recent years, increasing attention has been paid to the prevention of AF and associated complications through lifestyle and risk factor modification [4].

Several studies have explored the utility of exercise programs for the outcomes associated with AF. Programs focused on aerobic exercise and fitness, particularly when associated with weight loss, have been shown to improve arrhythmia burden and associated symptoms in patients with AF [5-7].

Cardiac rehabilitation (CR) is a supervised exercise program paired with health education. In the United States, CR is currently recommended for patients with a recent myocardial infarction, recent percutaneous coronary intervention or bypass surgery, cardiac valve surgery, or congestive heart failure. Several studies have investigated the effect of these programs on patients with AF, with generally favorable results for symptom reduction and arrhythmia control but mixed results for mortality and hospitalization [8-17].

CR is, however, practically limited by the lack of insurance coverage in the United States for patients with AF. Another limitation is the requirement for participants to physically report to gymnasiums or health care centers to participate in exercise sessions under the supervision of health care personnel. The resource intensiveness of these models limits their scalability, and in-person design limits accessibility for patients who may be working or live remotely from centers offering these services. As a result, it remains that CR as a therapy has a low penetrance, even among patients for whom it is currently indicated and covered. There is, however, growing interest in the evaluation of alternative models of delivery, strategies to improve access, and the evidence-based evolution of program design [18].

Our center has been offering remote CR services for patients indicated for traditional CR but unable to participate for personal reasons such as work or distance. In this pilot study, we sought to evaluate the feasibility of offering a 12-week asynchronous remotely supervised exercise program, modeled on CR programs, augmented with digital health tools and AF-specific educational material to patients with AF undergoing catheter ablation.

## Methods

### Patient Cohort

All patients referred for catheter ablation for AF at a tertiary care center were considered for enrollment. The inclusion criteria were (1) age 18 years or older, (2) access to a smartphone compatible with the provided digital health equipment, (3) access to exercise equipment or space, (4) low to moderate risk based on the American Association of Cardiovascular and Pulmonary Rehabilitation criteria, and (5) presence of 1 additional risk factor, including obesity (BMI >30 kg/m^2^), sedentary lifestyle, hypertension, diabetes mellitus, dyslipidemia, or obstructive sleep apnea. The exclusion criteria included (1) the inability to provide informed consent, (2) major procedural complications, (3) pulmonary disease requiring home oxygen, (4) gait instability, or (5) history of previous falls.

A total of 12 patients were enrolled in this pilot study. An additional 14 patients were screened but not enrolled, 12 of whom were male. A patient met exclusion criteria on further review. A total of 6 patients could not be reached for review of the study, and 2 canceled their procedures. Among the remaining 5 patients who declined, 1 was due to relocation and the remaining patients did not provide a specific reason.

### Intervention Design

All patients were provided with a weight scale, a blood pressure cuff, and a Kardia Mobile 6L electrocardiogram (ECG) device (AliveCor). All participants were enrolled in KardiaPro (AliveCor) for ECG review and storage. Patients were also enrolled in the patient portal of the electronic medical record (EMR), where the ability to enter self-recorded data was enabled with subsequent display in a patient-generated data flowsheet.

**Figure 1:**
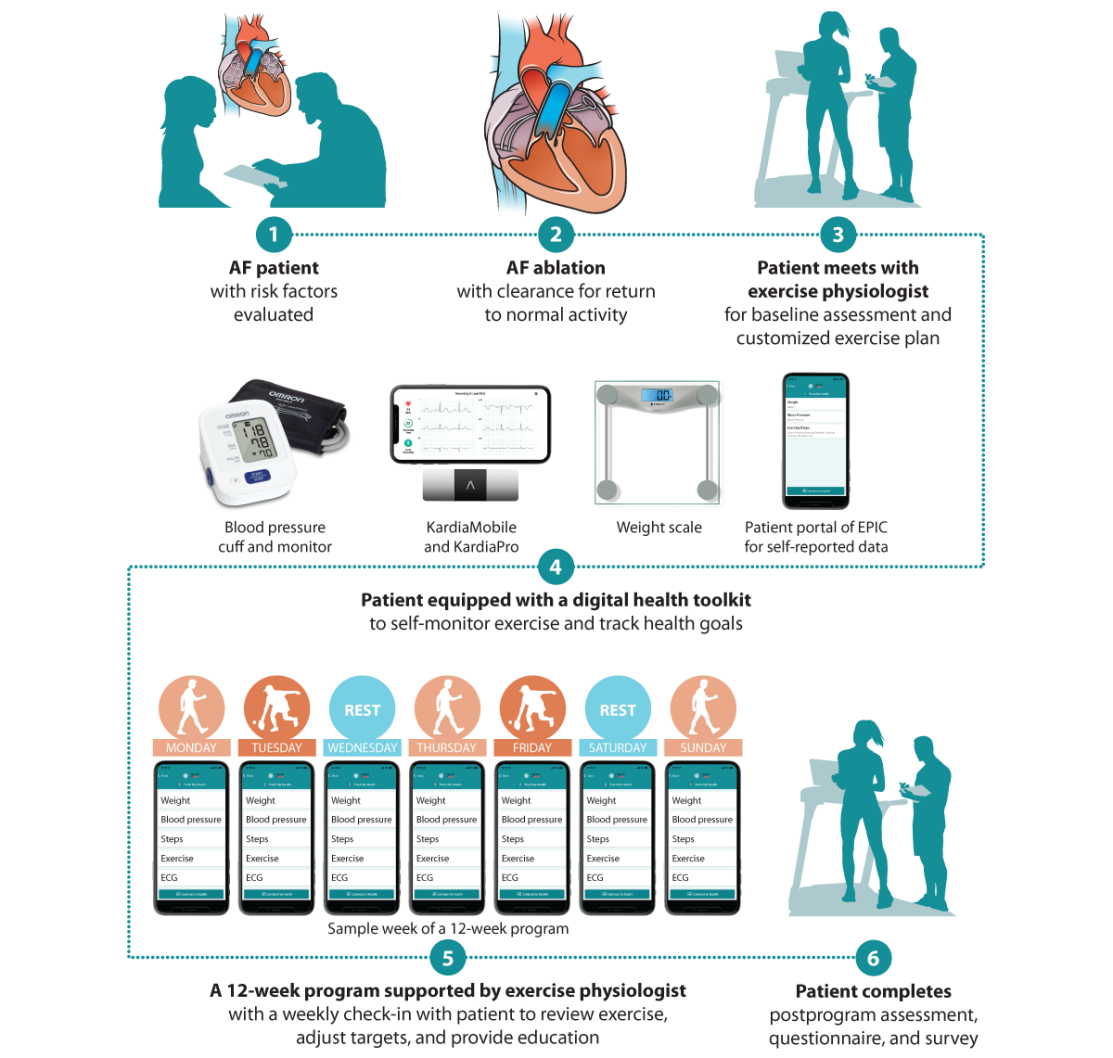
Overview of a remotely supervised exercise program for patients with atrial fibrillation (AF). ECG: electrocardiogram.

As summarized in Figure 1, all patients underwent a 1-week postablation follow-up visit with an electrophysiology advanced practice provider, at which point they were cleared for return to normal activity. An in-person intake visit was then scheduled for a visit with an exercise physiologist in the CR gymnasium, and the patient completed the Atrial Fibrillation Effect on Quality-of-Life Questionnaire.

During the intake visit, baseline weight, blood pressure, and waist circumference were checked. The patient then completed a 6-minute walk test (6MWT) on a treadmill or on the track. The exercise physiologist discussed a home exercise plan and fitness goals with each patient. The exercise physiologist also reviewed an exercise target heart rate range and rating of perceived exertion (RPE) to help guide their home exercise. Each patient was shown how to track exercise minutes, blood pressure, and weight to track their health within Epic (Epic Systems Corp), the electronic health record (EHR) system. If available, a participant’s Fitbit (Fitbit Inc) or Apple Watch (Apple Inc) was set up and synced with Epic to help with tracking. Each patient was given the opportunity to try a variety of aerobic equipment in the CR gymnasium. The personalized exercise program was modeled on the recommendations provided during standard CR programs, and a remote CR program at our institution was offered to patients who met the criteria for ambulatory CR programs but were unable to participate. During this visit, patient education was provided directly by the exercise physiologist and through print material regarding diet, exercise, and self-monitoring, such as techniques for appropriate blood pressure measurement.

The exercise physiologist made phone calls to follow up with patients on a weekly basis. The physiologist discussed (1) how often the patients were exercising, (2) how they were progressing, and (3) if they were having any symptoms. Patients were given an opportunity to ask questions about their home exercise routine. The exercise physiologist would review the data tracked in Epic and follow up with patients regarding their self-tracking. Exercise targets were adjusted by the exercise physiologist based on the data review, particularly the RPE, and discussion with the participant. The specific changes were not protocolized but included both duration and intensity adjustments in parallel when made. Throughout the 12-week intervention, additional education was provided through direct patient communication with the exercise physiologist and print materials.

During the program, patients were asked to record their weight and blood pressure on a daily basis and exercise minutes as performed; the data were recorded in the patient portal of the EHR. Participants were asked to record ECGs on a daily basis and with symptoms; the ECGs were reviewed and stored in KardiaPro. Patients with an iPhone (Apple Inc) had Apple Health (Apple Inc) sharing enabled them to allow automatic data flow from their devices into the EHR. After the completion of the 12-week program, patients were permitted to continue entering data at their discretion.

At the end of 12 weeks, patients participated in an exit evaluation, including measurements of weight, waist circumference, 6MWT, and Atrial Fibrillation Effect on Quality-of-Life survey (AFEQT) questionnaires. At least 3 months after completion of the program, participants were asked to complete a 4-question survey using a Likert scale on their experience and also to indicate interest in patient advisory roles for future program development and study. Both AFEQT and RPE have been validated in previous literature [19,20].

### Outcomes

The primary end point of this study was program completion, specifically participation through completion of the exit visit. Secondary end points included self-tracking adherence, weight, 6-minute walk test, waist circumference, change in AFEQT score, and program satisfaction survey. Finally, during the first 3 months of the study after enrollment of the first patient, the exercise physiologist time applied toward the program was recorded for the assessment of personnel requirements. Except for weekly patient tracking, all of the other patient data were collected and stored in an encrypted REDCap (Research Electronic Data Capture; Vanderbilt University) database. Patient self-tracking data were abstracted from the EMR and cloud-based ECG storage system for analysis.

### Statistical Analysis

Descriptive statistics were calculated for patients’ demographics and comorbidities and presented as mean (SD) or median (range) or counts (percentages), as appropriate. Outcome data were tested for the normal distribution using the Shapiro-Wilk test. A 2-tailed, paired *t* test for normally distributed data or a Wilcoxon rank sum test for not normally distributed data was performed to assess the differences between pre- and postintervention measures. Median change or mean change with SE were reported, as appropriate. All statistical analyses were performed using SAS Enterprise Guide 7.1 (SAS Institute). A *P* value of <.05 was considered statistically significant.

### Ethical Considerations

This study was reviewed and approved by the Atrium Health Institutional Review Board (IRB number: IRB00082393). All participants provided informed consent before being enrolled in the study. All protected health information collected was managed in accordance with institutional data protection protocols. Protected health information, including identifiable data, was stored on institutional servers secured according to institutional protocols. There was no compensation provided for participation in the study.

## Results

### Baseline Demographics

Baseline characteristics are summarized in Table 1. Half of the participants (6/12, 50%) were female, with a median age of 67.5 (range 47-79) years. The mean BMI was 33.8 kg/m^2^. Most of the patients had associated medical comorbidities. The median CHADs2VASC (Congestive Heart Failure, Hypertension, Age [≥75 years], Diabetes, Stroke/Transient Ischemic Attack, Vascular Disease, Age [65-74 years], Sex [Female]) was 1.5 (range 1-5). Slightly more than half of participants (7/12, 58%) had paroxysmal AF, while the remainder had persistent AF.

**Table 1:** Baseline characteristics of participants (N=12).

Participants		Frequency
Sex (female), n (%)		6 (50)
Race (White), n (%)		10 (83)
Age (years), median (range)		67.5 (47-79)
BMI (kg/m^2^), mean (SD)		33.78 (5.03)
**AF^a^ type, n (%)**		
	Paroxysmal	7 (58)
	Persistent	5 (42)
Diabetes, n (%)		2 (17)
Hypertension, n (%)		10 (83)
Heart failure, n (%)		1 (8)
Coronary artery disease, n (%)		2 (17)
Peripheral vascular disease, n (%)		1 (8)
Cerebrovascular accident, n (%)		1 (8)
CHADs2VASC^b^, median (range)		1.5 (1-5)
Obstructive sleep apnea, n (%)		6 (50)

^a^AF: atrial fibrillation.

^b^CHADs2VASC: Congestive Heart Failure, Hypertension, Age (≥75 years), Diabetes, Stroke/Transient Ischemic Attack, Vascular Disease, Age (65-74 years), Sex (Female).

### Program Adherence and Time Intensity

Program adherence and engagement with exercise physiologist is summarized in Table 2. In total, 92% (11/12) of participants completed the 12-week program through the exit visit. One patient was lost to follow-up during the program. Over the course of the 12-week program, 94% of expected patient contacts between the exercise physiologist and the participant were made, with a total of 136 patient contacts of an expected 144 contacts. The contact type, either telephone or email, was evenly distributed between email and telephone calls. The majority of contacts included educational content (123/136, 90%), while a smaller segment included support for self-tracking (48/136, 35%) and an adjustment of exercise targets (20/136, 15%).

For self-tracking, the cohort of 12 participants recorded 487 blood pressure measurements, 824 ECGs, and 418 exercise sessions over the 12-week program. This accounted for approximately 3.4 blood pressure measurements, 5.7 ECGs, and 2.9 exercise sessions per patient per week. For the requested daily recording of blood pressure and ECG, this frequency is associated with a 48% adherence rate and an 81% adherence rate, respectively.

There were no program-related adverse events. One patient developed COVID-19 during the program and had to pause participation.

**Figure 2:**
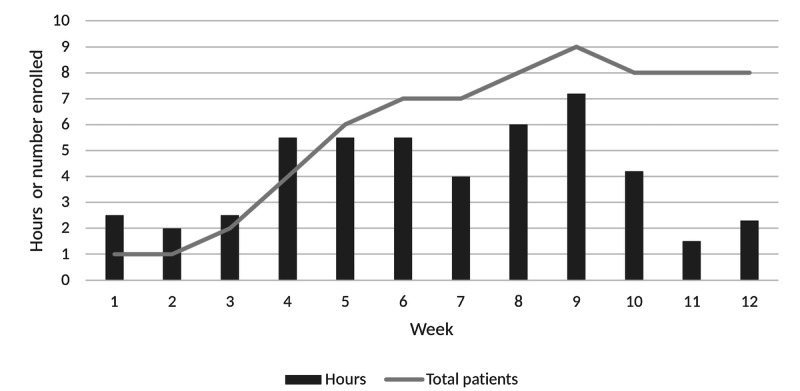
Exercise physiologist time use.

In the 3 months of the study, the period in which exercise physiologist time was specifically tracked, a total of 48.7 hours of exercise physiologist time was required. The number of patients enrolled in the study and time per biweekly period are shown in Figure 2. The average time per week required was 2 hours, or an average of 0.5 hours per patient enrolled per week.

**Table 2:** Participant adherence to self-tracking and weekly contacts with the exercise physiologist.

Patient-reported metrics				Total		Frequency
Blood pressure, median (range)				537		49 (0-83)
Electrocardiogram, median (range)				824		72.5 (32-92)
Exercise sessions, median (range)				418		36 (0-69)
**Participant contacts**						
	**Total contact**			136		94%^a^
		Phone	66		45%^a^	
		Email	70		49%^a^	
**Contact content**						
	Education			123		90%^b^
	Self-tracking			48		35%^b^
	Exercise targets			20		15%^b^

^a^Percentage expected.

^b^Percentage total.

### Health Outcomes and Patient Satisfaction

Program outcomes are summarized in Table 3. Overall, there were significant improvements seen across all measured domains. The average waist circumference reduced from 45.5 to 44.0 (*P*=.001). Weight reduced from 215.8 lbs to 211.94 lbs (*P*=.04). BMI also improved from 34.1 to 33.4 kg/m^2^ (*P*=.04). The 6-minute walk tests also improved from 0.31 km to 0.38 km (*P*=.01). AF symptoms, as assessed by the AFEQT questionnaire, also improved from 40.6 to 80.0 *(P*<.001).

The postprogram surveys were returned by 8 participants (Table 4). Among the participants responding, the program assessment was favorable. Participants overall strongly agreed that the program improved their overall health and wellness (8.75/10). They also strongly agreed that they would recommend this program to other patients with AF (9.13/10). Finally, they strongly agreed that they would be able to apply the education they received to future decisions about their health and that they could sustain the established exercise program going forward (9/10 and 8.215/10, respectively).

There were no intervention-related adverse events. One patient developed COVID-19 during the intervention, requiring an interruption of exercise.

**Table 3:** Health outcomes of a remotely supervised exercise program.

	Preprogram measurement, mean (SD)	Postprogram measurement, mean (SD)	Absolute change, mean (SD)	*P* value
Waist circumference (inches)	45.5 (4.9)	44 (4.4)	1.5 (1.2)	.002
Weight (lbs)	217.8 (36.1)	213.3 (33.5)	4.6 (6.2)	.04
BMI (kg/m^2^)	34.1 (5.2)	33.4 (5.2)	0.7 (1)	.03
6-minute walk test (km)	0.3 (0.1)	0.4 (0.1)	–0.1 (0.1)	.02
AFEQT^a^ score	40.6 (21.2)	80.1 (17.3)	–39.5 (20.6)	<.001

^a^AFEQT: Atrial Fibrillation Effect on Quality-of-Life survey.

**Table 4:** Patient feedback on a remotely supervised exercise program.

Question (1=strongly disagree and 10=strongly agree)	Mean score
This program improved my overall health and wellness	8.75
I would recommend this program to other patients with atrial fibrillation	9.125
I will be able to apply the education to my future decisions about exercise and health	9
I will be able to sustain the exercise program I established going forward	8.125

## Discussion

In this study of an asynchronous remotely supervised exercise program augmented with disease-specific education in patients undergoing catheter ablation of AF, we found that there was a high rate of program adherence without significant program-associated adverse events and high levels of patient satisfaction.

Several studies have investigated the utility of exercise programs for patients with AF. In the CopenHeartRFA study, Risom et al [15] randomized 210 patients undergoing catheter ablation for AF to CR versus usual care. While a significant difference in peak oxygen consumption was found, there was no difference in quality of life as assessed by the 36-Item Short Form Health Survey questionnaire [15]. Kato et al [10] observed similar findings in their study of 61 patients randomized to CR versus usual care. In the extended follow-up of this cohort, Risom et al [16] reported persistence of this effect at 1 year as well as lower levels of anxiety in the intervention cohort at 2 years, though no differences in hospitalization or mortality were observed. Aoyama et al [9] evaluated the effect of CR among patients with heart failure undergoing catheter ablation of AF. In that study, no significant outcome difference was observed over 18 months of follow-up in AF recurrence or hospitalization. Overall, data specifically on the impact of CR are limited and conflicting in terms of impact on arrhythmia burden and outcomes [13,14].

Other studies have more generally evaluated risk factor modification programs, including components focused on exercise. In the CARDIO-FIT study, 308 patients were enrolled in a risk factor modification program that included elements focused on comorbidities including obesity, hypertension, glucose intolerance, and sleep apnea. Participants underwent exercise stress testing, and a tailored exercise program was designed for each participant. Participants with >2 metabolic equivalent improvement in fitness had the greatest arrhythmia-free survival at follow-up (89% vs 40%, *P*<.001). Interestingly, participation in a separate risk factor clinic was strongly associated with the achievement of improvement in cardiorespiratory fitness (83% vs 39%, *P*<.001), suggesting significant value for reinforcement and observation from health care providers. Patient education has also been demonstrated to be an important component in the care of patients with AF. In HELP-AF, Gallagher et al [21] evaluated an in-home educational program including an educational pamphlet designed with patient feedback and demonstrated a reduction in subsequent AF hospitalizations. Isakadze et al [22] recently described the development of educational material delivered through a smartphone app using patient-centered design processes as well, which could offer a far more scalable approach to patient education.

In comparison with these previous investigations, this study offers several strengths. First, the use of digital care models with digital health tools, patient-reported data through EMR interfaces, and remote follow-up can help improve scalability and accessibility. Several factors, including the distance from appropriate facilities and socioeconomic barriers, have been identified as contributing to the poor rates of participation in CR among eligible patients [23,24]. Second, the inclusion of remote monitoring of participation could help improve adherence. As Pathak et al [6] noted, there was a substantial improvement in the impact of their exercise intervention among patients who had participated in a risk factor clinic. Similarly, weekly check-ins with an exercise physiologist associated with the arrhythmia team may help motivate patients and improve the effectiveness of the intervention. Additionally, pairing with the periablation period may help leverage routine patient contacts to reinforce the importance of exercise in AF management. Finally, the time requirement for staff engagement, specifically the exercise physiologist, was low per patient and may help improve the feasibility of implementation in a health care environment where staffing is more challenging.

There are several limitations worth noting. First, the sample size was small, and so all findings should be viewed as hypothesis generating and informative for future investigations. A contributing factor to the small sample size was several COVID-19 surges, which led to the cessation of research enrollment for extended periods. Nonetheless, this pilot study provided several insights into future iterations and further studies, such as staffing requirements, participant self-monitoring adherence in several areas, and technological gaps to be addressed. Also, it was a homogeneous cohort, and future studies will need to focus on improvement in size and diversity of the enrolled population. A structured assessment of health literacy, technological sophistication, and disease-specific knowledge would also be useful to help assess the intervention’s impact and the generalizability of results.

Second, effects on AF-associated symptoms are confounded by the postablation status of the cohort. While mitigated somewhat by the baseline AFEQT being done several weeks after catheter ablation, the effect on AF symptoms would be better assessed with a control group. Additionally, while ECG monitoring was performed during the study, there was neither a specific recommendation to check before exercise nor a restriction that exercise should not be performed if in AF. Furthermore, while heart rate targets were provided, these were not adjusted for rhythm, so it is possible that participants limited exercise based on elevated heart rates in AF if they occurred during exercise. In future iterations of program design, rhythm assessment with the adjustment of rate targets accordingly would be an important consideration. Finally, the collection of physiologic data during the intervention was primarily exploratory to assess adherence and feasibility. No conclusions can be made in regard to the impact on those measures from the intervention.

There are several observations that were noted through the course of this pilot study that will help inform future intervention design. First, 50% of potential candidates, excluding the 2 patients who canceled their procedure and therefore were no longer candidates, were enrolled in the program. For most patients who declined to participate, no specific reason or barrier beyond preference was identified. It is also important to recognize that disparities exist in patient referrals for catheter ablation for patients of certain minority groups [25]. As such, interventions targeted at patients reaching this stage will inherit these disparities, and expansion beyond this cohort will be important to ensure equitable access.

Second, a more robust data aggregation infrastructure will be required. The embedded functionality for patient-generated data within the EMR performed poorly due to (1) poor visualization tools, (2) system stability issues with large quantities of data, and (3) lack of tools for data export for analysis requiring manual abstraction. Functionality for exercise tracking beyond patient self-report was limited as well, despite the availability of more robust activity tracking tools. For such programs to be feasible, a single data management and software solution will be critical to ensuring the efficiency and scalability required to clinically implement this type of program. Third, some patients expressed a preference for access to the exercise facilities at the CR center, even outside of the directly supervised programs currently available but for which they were not candidates.

There are several opportunities to improve the scalability of this type of intervention. Automated tools for longitudinal tracking of patients will be needed to support the durability of the changes made during the intervention. Additionally, protocol-driven feedback and adjustments to exercise regimens based on the collected data could help improve outcomes; further validation of these protocols, however, would be important. Lastly, the need for an in-person visit to a CR center may be a barrier both in terms of access and cost; future iterations will need to explore alternative models for program intake and exercise prescription development.

In summary, this asynchronous remotely supervised exercise program augmented with AF-specific educational components for patients with AF appears feasible in terms of patient adherence as well as resources required for implementation and warrants further iterative development and study. Further iteration of data collection strategies, including both implementation of aggregation tools outside the EHR and more focused self-monitoring recommendations, will need to be developed. Particular attention to the recruitment of diverse cohorts of patients for future studies will be important to evaluate strategies to overcome barriers that may be presented by socioeconomic, racial, geographic, and technological disparities. In comparison to traditional care paradigms currently available, this type of intervention can offer scalable strategies for risk factor modification across large patient cohorts dispersed across broad geographic areas and warrants further investigation.

## Abbreviations

6MWT: 6-minute walk test
AF: atrial fibrillation
AFEQT: Atrial Fibrillation Effect on Quality-of-Life survey
CHADs2VASC: Congestive Heart Failure, Hypertension, Age (≥75 years), Diabetes, Stroke/Transient Ischemic Attack, Vascular Disease, Age (65-74 years), Sex (Female)
CR: cardiac rehabilitation
ECG: electrocardiogram
EHR: electronic health record
EMR: electronic medical record
REDCap: Research Electronic Data Capture
RPE: rating of perceived exertion

## Data Availability

The data sets generated or analyzed during this study are available from the corresponding author upon reasonable request.
